# Probable RBD Associates with the Development of RLS in Parkinson's Disease: A Cross-Sectional Study

**DOI:** 10.1155/2019/7470904

**Published:** 2019-04-01

**Authors:** Yewei Qu, Lu Zhang, Dongfang Shen, Wangzikang Zhang, Mingsha Zhang, Yujun Pan

**Affiliations:** ^1^Department of Neurology, The First Hospital of Harbin Medical University, Harbin, China; ^2^Department of Neurology, Daqing Oilfield General Hospital, Daqing, China; ^3^Department of Neurology, The Fourth Hospital of Harbin Medical University, Harbin, China; ^4^Zuckerman Mind Brain Behavior Institute, Columbia University, New York, USA; ^5^State Key Laboratory of Cognitive Neuroscience and Learning, Beijing Normal University, Beijing, China

## Abstract

**Objectives:**

We aimed to investigate the prevalence of restless leg syndrome (RLS) and exploring the contributing factors that affect the development of RLS in Parkinson's disease (PD) patients.

**Methods:**

A cross-sectional study was conducted consisting of 178 consecutive PD patients from our hospital between October 2015 and August 2016. We divided the participants into two groups, which were PD with RLS and PD with non-RLS. Then, we recorded their demographics and clinical data to draw a comparison between PD with RLS and PD with non-RLS.

**Results:**

23 (12.92%) were diagnosed with RLS among all the enrolled PD patients. Unified Parkinson's Disease Rating Scale III (UPDRS III) and Hamilton Depression Scale (HAMD) scores, probable rapid eye movement sleep behavior disorder (PRBD), and daily levodopa equivalent dose (LED) in the PD with the RLS group were significantly different from those in the PD with the non-RLS group. Daily LED and the scores of UPDRS III and HAMD in PD patients with RLS were all higher than those in PD patients with non-RLS. PRBD, daily LED, and HAMD scores were significantly independent factors contributing to the development of RLS (OR = 4.678, 95% CI 1.372~15.944, *P* = 0.014; OR = 1.003, 95% CI 1.001~1.005, *P* = 0.019; OR = 1.094, 95% CI 1.002~1.193, *P* = 0.045). The severity of RLS was positively correlated with the duration of PD and daily LED (*r* = 0.438, *P* = 0.036; *r* = 0.637, *P* = 0.001).

**Conclusion:**

PRBD existence, daily LED, and HAMD scores are independent factors for developing RLS in PD patients. PRBD existence is firstly proposed as an independent factor in developing RLS among PD patients. RLS severity in PD patients are positively associated with the duration of PD and daily LED.

## 1. Introduction

Parkinson's disease (PD) is a neurodegenerative syndrome involving multiple motor and nonmotor symptoms. Besides the major motor symptoms, PD's nonmotor symptoms have been widely studied over the past decades. Sleep disorders are the most common among PD's nonmotor symptoms [1]. Restless leg syndrome (RLS) is a common sleep-related movement disorder characterized by an urge to move the limbs frequently accompanied by uncomfortable and unpleasant sensations that are difficult to describe [2]. A growing number of literatures investigated the association between RLS and Parkinson's disease in recent years, while some issues are still disputable. For example, impaired dopaminergic pathway may represent a bridge between these two conditions or if RLS represents a secondary condition of PD. Moreover, many factors, including age [3–8], sex [4, 9], duration of PD [10, 11], PD severity [4, 11–13], psychiatric symptoms [5, 9, 13–15], cognition [4, 11, 13], dyssomnia [9, 12–14, 16], serum ferritin level [3], dopaminergic treatment [7, 11], and autonomic dysfunction [13], can influence the development of RLS in PD patients. Quantities of researches having been done on the potential interrelation between PD and RLS demonstrated the overlaps existing between PD and RLS, including genetic background [3, 4, 11, 17], efficiency of dopamine agonist treatment, and dysfunction of the central dopaminergic system in pathophysiology [3, 10, 11, 17].

In this study, we aimed to study the prevalence of RLS in Chinese PD patients and search for some other possible unidentified factors associated with the development or severity of RLS.

## 2. Materials and Methods

### 2.1. Participants

This observational study was conducted at the Neurological Department of the First Hospital of Harbin Medical University from October 2015 to August 2016. All patients were freely given informed consent to participate in this research project.

All participants fulfilled the 2015 Movement Disorder Society clinical diagnostic criteria for PD [18]. The participants had no surgical history of deep brain stimulation or pallidotomy and no history of anxiolytic drugs or antidepressants in the past few months. They also showed normal levels of serum iron/serum ferritin/total iron-binding capacity and no seriously systemic diseases. Patients were excluded if they had atypical parkinsonism.

The RLS diagnosis was made with face-to-face interviews and questionnaires which were done by the same neurologist and follows the International RLS Study Group (IRLSSG) criteria revised in 2014: (1) an urge to move the legs, usually accompanied or caused by an uncomfortable sensation in the legs, (2) the beginning or worsening of symptoms during periods of rest or inactivity, (3) the partial or total relief of symptoms by movement, (4) the symptoms being worse in the evening or night than during the day or only occurring in the evening or night, and (5) the occurrence of the above features is not solely accounted for as symptoms primary to another medical or a behavioral condition (e.g., myalgia, venous stasis, leg edema, arthritis, leg cramps, positional discomfort, and habitual foot tapping) [19]. A positive diagnosis of RLS was made when a patient had all five symptoms above.

### 2.2. Study Design

We collected all patients' clinical and demographic information (including sex, age, age of PD onset, duration of PD and RLS, family history of PD and RLS, severity of PD, and medication treatments for PD). In order to facilitate the analysis, the total daily levodopa equivalent dose (LED, mg/day) was calculated according to the previously established methods, by which 100 mg of levodopa is equal to 130 mg of levodopa in a controlled released form, 70 mg of levodopa if also using entacapone, 1 mg of pramipexole, 5 mg of ropinirole, 5 mg of rotigotine, and 100 mg of piribedil [4].

Motor examination scores from the Unified Parkinson's Disease Rating Scale III (UPDRS III) were obtained on drug treatments for PD to assess the patients' motor disability. With regard to the PD and RLS severities, we based the evaluations on the Hoehn and Yahr (H-Y) stage and the Restless Leg Syndrome Rating Scales (RLSRS). Participants also completed the Minimum Mental State Examination (MMSE) and the Hamilton Depression Scale (HAMD) questionnaires to, respectively, assess cognitive function and the presence of depression. Probable rapid eye movement sleep behavior disorder (PRBD) was assessed by the RBD questionnaire response (RBDSQ) [6] via a patient self-rating questionnaire. The score, which was equal or greater than six, was considered as PRBD in this study [20].

All participants were divided into two groups, which were PD with RLS and PD with non-RLS. The PD patients with RLS were further subdivided into two groups based upon the sequence of RLS and PD motor symptoms.

### 2.3. Statistical Analysis

Data were analyzed using Statistical Product and Service Solutions version 22.0 (SPSS Inc., Chicago, IL) software package. Categorical parametric data were presented as percentages. Continuous parametric data satisfying normal distribution were described as mean ± SD. If the data were nonnormally distributed, we chose chi-square test for categorical variables and described as median values with their corresponding interquartile. With regard to the continuous variables, *t*-test was used when the data fell under normal distribution and variance homogeneity. If normal distribution was not fulfilled, the Mann–Whitney *U* test was adopted. If a linear relationship matched the normal distribution, correlation analysis of the data was conducted with the Pearson correlation test. If all above were unsatisfied, the Spearman correlation test was adopted instead. Logistic regression analysis was performed to test significantly independent risk factors for developing RLS in PD patients. We tested the statistical significance hypothesis by the likelihood ratio test with an *α* value of 0.05. We reported all associations as odds ratios with their corresponding 95% CIs.

## 3. Results

In total, 178 patients who complied with the inclusion and exclusion criteria were recruited into this study.

### 3.1. Prevalence and Clinical Features of RLS in PD Patients

Of the total participating patients, 23 (12.92%) suffered from RLS with all of them having no family history of RLS or PD. Among all the RLS patients who recalled their progression of symptoms, sixteen showed PD symptoms prior to RLS, and the rest appeared later. In the latter seven cases, the RLS symptoms of five patients disappeared after taking antiparkinsonian medications. Nevertheless, the remaining two showed that from 5 to 7 years of treatments with antiparkinsonian medications, their RLS symptoms initially disappeared and then reappeared later on. Moreover, compared to the previous five patients, these two patients spent more years on antiparkinsonian medication treatments until RLS symptoms disappeared. Additionally, symptoms in these seven patients begun from the right side, with five out of these seven presenting tremors as the first symptom. Compared with RLS prior to the PD group, symptoms involved bilateral sides in the majority among patients with PD prior to PLS (12/16, 75%). Only one of the patient's symptom involved bilateral sides in RLS prior to the PD group. In RLS prior to the PD group, the interval between RLS and PD varied from about 7 to 18 months, and in PD prior to the RLS group, it ranged from 2 to 10 years.

### 3.2. Comparison of Clinical Data between the PD with the RLS Group and the PD with the Non-RLS Group

The clinical data comparing the two groups are shown in Table 1. Significant differences were observed between the two groups in PRBD, daily LED, and UPDRSIII and HAMD scores by one-way analysis of variance. Daily LED and the scores of UPDRS III and HAMD in PD patients with RLS were all higher than those in PD patients with non-RLS. However, no significant differences were found between these two groups for other factors, such as sex, age, age of PD onset, duration of PD, unilateral/bilateral involvement, grade of H-Y, and MMSE score.

### 3.3. Factors Contributing to the Development of RLS in Patients with PD

Comparing the clinical data between the PD with the RLS group and the PD with the non-RLS group (Table 1), we selected PRBD, daily LED, and UPDRS III and HAMD scores as independent variables, whether existing RLS served as a dependent variable for multivariate logistic regression analysis. The results in Table 2 showed that PRBD, daily LED, and HAMD scores were significantly independent factors contributing to the development of RLS in PD patients.

### 3.4. Analysis of Relative Factors of RLS Severity

The severity of RLS in PD patients was positively correlated with duration of PD and daily LED. RLS severity had no correlation with sex, age, age of PD onset, unilateral/bilateral involvement, UPDRS III score, H-Y grade, PRBD, MMSE score, and depression (Table 3).

## 4. Discussion

It has been widely recognized that RLS is more likely to occur among PD patients [3, 5, 7, 11, 12, 14, 21]. A meta-analysis of 11 studies showed chances of RLS prevalence among PD patients being nearly threefold higher than healthy controls [22]. This analysis also suggested an overall RLS prevalence of 14% in PD patients based on IRLSSG diagnostic criteria. The prevalence appeared to be slightly higher outside Asia (16%) than within Asia (12%) [22]. Our study demonstrated that among PD patients, the prevalence of RLS was 12.92%, which is consistent with the overall level. Nevertheless, the prevalence of RLS in PD patients was documented with high variability in different studies [10, 23]. Ethnicity, geographic areas, and research methods are likely to explain at least part of this discrepancy [2, 22].

In previous studies, onset of RLS in relation to onset of PD was evaluated several times. Rijsman et al. stated that 76% to 100% of patients showed an onset of RLS only after or together with the onset of PD [24]. One research found that, for PD patients with a positive RLS family history, the RLS onset preceded the PD onset more often (52%) than for PD patients without a positive RLS family history (29%) [3]. RLS symptoms preceded PD symptoms in 15% of all patients. Thus, they speculated that the range of neuropathological lesions of PD played a decisive role in the presence of RLS. Among all the RLS patients in our study who recalled their progression of symptoms, seven patients appeared RLS prior to PD. In view of family histories that relied on subjective recall only, we have reasons to suspect that the seven participants are more likely to have family histories than the remaining sixteen. After all, some pathogenesis which causes RLS probably have little association with PD, while it cannot be recognized clinically. For instance, a report demonstrated that known susceptible genes for idiopathic RLS such as MEIS1, BTBD9, and MAP2K5-LBXCOR1 [25] were not associated with PD [26]. Besides, even if PD have been treated as a secondary reason for RLS, RLS could also be a comorbidity of PD. In our study, approximately 30% of our patients had RLS symptoms prior to PD onset, which was higher than that in previous studies [3]. This may have been due to our study's small sample size.

Additionally, the RLS symptoms of these seven cases of patients disappeared after taking different combinations of antiparkinsonian medications, including levodopa, dopamine agonists (pramipexole), and monoamine oxidase B inhibitor (selegiline). We speculate that to some extent, RLS and PD might share similar pathophysiological overlaps since antiparkinsonian treatment is effective for both. However, it is still under debate on whether or not they share a common pathophysiology [22]. Moreover, after taking antiparkinsonian medications, the RLS symptoms in two of these seven initially disappeared while 5 and 7 years later, RLS reappeared. Moreover, compared to the five patients, these two spent more years on antiparkinsonian medication treatments until the original RLS symptoms disappeared. The reappearances of these two tend to be “true” RLS rather than mimic RLS on the basis of distinguishing features. For instance, even if the differences between the motor restlessness in akathisia and RLS is subtle, the motor restlessness in akathisia is usually a generalized whole-body sensation, without sensory discomfort, a strong circadian rhythm, and relief by movement. The “true” RLS seemed to have a negative impact on the sleep quality. PD patients with RLS might have more sleep disturbances, as indicated by the responses on the following subitems: “nocturnal restlessness of legs or arms at night or in the evening, causing sleep disruption,” “numbness or tingling of arms or legs with an awaken effect,” “fidgeting in bed,” and “painful muscle cramps in arms or legs whilst sleeping at night” [24]. Also, the urge to move the legs is usually not part of PD symptomatology [27]. As a matter of fact, RLS symptoms are similar to akathisia, dystonia, and some sensory symptoms of PD [11]. Long-term dopaminergic therapy can induce symptoms similar to RLS [11], and RLS-like symptoms may be the features of the “wearing off” phenomenon [17]. Interestingly enough, certain leg motor restlessness symptoms that do not fulfill all RLS criteria can later develop into full-blown RLS in PD patients [28]. During an 8-year follow-up of 3.5 million US veterans, Szatmari et al. [29] found that RLS could be an early clinical feature of PD. Thus, exactly as assumed by Ferini-Strambi et al., a twofold interpretation needs to be taken into account: dopaminergic therapy may have a crucial role in the development of RLS in PD patients or RLS can be conceived as an early manifestation of PD rather than a risk factor [2]. In addition, dopaminergic medication is a conventional treatment for PD and has a therapeutic effect on RLS. Therefore, it may hide potential RLS symptoms. Kedia et al. [30] has confirmed that a latent RLS was seen to “emerge” in PD patients who withdrew dopaminergic drugs after undergoing deep brain stimulation surgery targeted at the subthalamic nucleus. Furthermore, to date, no specific tool for assessment of RLS in PD exists, and whether the IRLSSG criteria is suitable for RLS in PD patients is controversial [3, 9]. These above could possibly have impacts on the assessment of RLS prevalence in PD patients.

We naturally ought to discuss the relation between dopaminergic treatment and RLS among PD patients. One research found that dopaminergic treatment duration was a significant predictor of the presence or absence of RLS [11]. The authors stated that the more frequent RLS in PD patients with longer dopaminergic treatment could be the result of the same mechanisms as those which induce an augmentation of RLS in prolonged levodopa exposure [11]. Dopaminergic augmentation is described as a spreading of symptoms to other body parts, a time shift in the start of symptoms to earlier in the day, a shorter latency to RLS symptoms at rest, and an increase in the severity of RLS [31]. Although the augmentation mechanisms are not fully clarified until now, the article demonstrated that the augmentation could be a consequence of overstimulation by the dopaminergic medication of the D1 dopamine receptors compared to the D2 receptors in the spinal cord [24]. The subsequent study found that after subthalamic stimulation concomitant with dopaminergic medication dose reduction, the RLS severity was decreased. This further endorses the hypothesis that RLS in PD can be a result of dopamine receptor overstimulation by dopaminergic medication [32]. Therefore, the augmentation might be one of the possible explanations to the reappearance of RLS for these two patients.

Not only dopamine treatment duration but also the dopaminergic medication dose was regarded as a factor that might influence the dopaminergic neurons. A study showed that higher dopaminergic medication might lead to overstimulation of dopaminergic neurons [11]. Angelini et al. [33] also certified the relevance between the occurrence of RLS in PD patients and dopaminergic medication therapy. Our study revealed that both the presence and severity of RLS were positively correlated with daily LED and that daily LED may be an independent factor for RLS development. But Peralta et al. [7] argued that the presence of RLS was negatively correlated with daily LED and suppress the RLS symptoms. This hypothesis was supported by Verbaan et al. [13], who issued the possibility that the relatively high mean dopaminergic dose could result in an underestimation of RLS patients. In addition, Kedia et al. [30] observed 11 RLS patients in 195 PD patients after subthalamic stimulation with a concurrent 75% reduction of the mean dopaminergic medication. Up to now, it is still difficult to find a point of agreement about the correlation between studies. Hence, the research between the dopaminergic medication dose and RLS should be further explored to help guide clinical treatments.

We deemed a relatively complicated interaction between depression and RLS. In our research, PD patients with RLS got higher HAMD scores than those with non-RLS, which was aligned with several past research perspectives [5, 12, 21]. Verbaan et al. [13] also raised that RLS severity was positively correlated with depression. In fact, depression and antidepressants (serotonin reuptake inhibitors) have been proposed to promote the progress of RLS and periodic limb movements in sleep [34]. Moreover, studies have shown that the potential mechanism of depression in PD-RLS patients was the dysfunction of the 5-HT system [35]. Piao et al. [36] furtherly observed that the 5-HT level in CSF in PD with the RLS group was significantly lowered compared with that in PD with the non-RLS group, and they supposed that a dysfunction of the 5-HT system might be a potential neurochemical mechanism underlying depression in PD patients with RLS. However, the potential mechanism of depression in PD patients with RLS remains unclear.

Our research revealed that PRBD might be regarded as a significantly independent factor which could influence the development of RLS. Only Ylikoski et al. [1] put forward that RLS is associated with the presence of RBD without a clearer explanation. In this article the authors stated sleep bruxism (SB), a parafunctional activity during sleep that is characterized by clenching (tonic activity) and/or the repetition of phases of muscle activity (phasic activity) that produce grinding of the teeth, and other isolated sleep symptoms were all significantly related to RBD. Moreover, 86.4% of the PD patients with RBD had at least one other parasomnia or some isolated sleep symptoms. That is to say, RBD coexisting without other parasomnias or isolated sleep symptoms was rare. As the decreasing prevalence of tooth grinding with age, SB is not associated with PD but perhaps more likely with RLS [37, 38]. These above perhaps prompt a potential link between RBD and RLS, especially among PD patients. While, so far, the relationship between RBD and RLS has been little studied systematically. There might be an inner link between PD, RLS, and RBD that deserve to be further explored.

Iron is an important cofactor in dopamine metabolisms and can also produce neurotoxic species [2]. Several studies have implicated both the dopaminergic system and the iron in PD and RLS, thus suggesting a probably common physiopathological basis. Nonetheless, the data showed in particular a depletion of DA in PD and a hyperdopaminergic state in RLS, which is inconsistent with the theory [2]. In PD, neurodegeneration occurs mainly in the substantia nigra (SN) [39]. A research found that the iron in PD patients in the SN was 1.5 times as much as the control group iron [40]. But many studies have verified a significant reduction of iron and ferritin in the SN of RLS patients by immunohistochemical staining [41], imaging [42], or cerebrospinal fluid (CSF) test [43]. In our study, all the enrolled patients had normal levels of related indicators for iron metabolism in the blood, including serum iron, serum ferritin, and total iron-binding capacity. Yet, it is a disputable issue whether there is a remarkable difference of serum iron and ferritin that is because previous studies found no significant differences in serum ferritin levels between PD with RLS and PD with non-RLS [27, 44]. However, iron levels in serum in PD with the RLS group were lower compared with that in PD with the non-RLS group [36]. It is worth mentioning that related indicators for iron metabolism in CSF consistently showed obvious differences in some studies, implying a potential relationship between brain iron dysfunction and PD with RLS [36, 45, 46]. The nigrostriatal pathway is one of the main regions of iron deposition in brain [47]. Excessive iron deposition in the nigrostriatal pathway might cause iron insufficiency in the RLS-related regions, so that it might be relevant to PD patients with RLS. Therefore, the levels of CSF iron and ferritin, which might be treated as related indicators for iron metabolism in PD patients, can be more dependable than those of serum.

### 4.1. Limitations of the Study

Study limitations are acknowledged. RLS mimics, such as akathisia, end-of-dose deterioration with sensory symptoms, nocturnal dystonia, or painful neuropathy, were eliminated with rigorous application of the essential criteria questions and subsequent face-to-face interviews. Dopaminergic medication is an effective treatment for both PD and RLS, so that it may hide potential RLS symptoms. Whether the IRLSSG criteria is suitable enough for RLS in PD patients is uncertain. Hence, from an objective perspective, we could not rule out some RLS mimics completely, and further investigation is essential. Additionally, a majority of the clinical data was highly dependent on patients' interviews, but historical information is always subject to recall bias. Another point that the levels of CSF iron and ferritin were reorganized as a remarkable factor among PD patients whether with RLS or not, whereas these factors were not taken into consideration in our study.

## 5. Conclusion

Our present results support an inner link between RLS and PD in Chinese patients. PRBD existence, daily LED, and HAMD scores are confirmed to be independent factors for developing RLS in PD patients. PRBD existence is firstly proposed as an independent factor in developing RLS in PD patients. RLS severity in PD patients is positively correlated with duration of PD and daily LED.

## Figures and Tables

**Table 1:** Comparison of clinical data between the PD with the RLS group and the PD with the non-RLS group.

Variables	PD with RLS (*n* = 23)	PD with non-RLS (*n* = 155)	Test value	*P* value
Sex (*n* (%))			0.022	0.881^a^
Male	14 (60.87)	76 (49.03)		
Female	9 (39.13)	79 (50.97)		
Age at presentation (yr, $\bar{\text{x}}\pm \text{s}$)	65.22 ± 8.60	64.34 ± 9.58	0.417^t^	0.677^b^
Age of PD onset (yr, $\bar{\text{x}}\pm \text{s}$)	61.26 ± 8.73	60.89 ± 9.19	0.182^t^	0.856^b^
Duration of PD (yr, M(Q_R_))	5.00 (3.00)	3.00 (4.00)	1452.00	0.149
Side involved (*n* (%))			0.202^a^	0.653^a^
Unilateral	10 (43.48)	76 (49.03)		
Bilateral	13 (56.52)	79 (50.97)		
UPDRS III (M(Q_R_))	22.00 (13.00)	18.00 (13.00)	1148.00	0.006
Hoehn and Yahr stage (M(Q_R_))	2.00 (1.00)	2.00 (1.50)	1465.50	0.160
PRBD (*n* (%))				0.012^c^
With PRBD	19 (82.61)	84 (54.19)		
With non-PRBD	4 (17.39)	71 (45.81)		
MMSE score (M(Q_R_))	28.00 (4.00)	27.00 (3.00)	1644.50	0.545
HAMD score (M(Q_R_))	12 (13.00)	7.00 (8.00)	1023.00	0.001
Daily LED (mg/day, M(Q_R_))	437.50 (350.00)	300.00 (287.50)	962.00	≤0.001

PD = Parkinson's disease; RLS = restless leg syndrome; UPDRS III = unified Parkinson's disease rating scale III; PRBD = probable rapid eye movement sleep behavior disorder; MMSE = minimum mental state examination; HAMD = Hamilton depression scale; daily LED = daily levodopa equivalent dose. ^a^For *χ*^2^ test, ^b^for *t* test, ^c^for Fisher's exact test, and the rest is *U* test; *P* < 0.05 in PRBD, daily LED, UPDRS III, and HAMD scores.

**Table 2:** Multivariate logistic regression analysis of factors for PD patients with RLS.

Variables	Regression coefficient	OR value	95% CI	*P* value
UPDRS III	0.031	1.032	0.982~1.084	0.212
PRBD	1.543	4.678	1.372~15.944	0.014^∗^
HAMD score	0.089	1.094	1.002~1.193	0.045^∗^
Daily LED	0.003	1.003	1.000~1.005	0.019^∗^

PD = Parkinson's disease; RLS = restless leg syndrome; UPDRS III = unified Parkinson's disease rating scale III; PRBD = probable rapid eye movement sleep behavior disorder; HAMD = Hamilton depression scale; daily LED = daily levodopa equivalent dose; OR > 1 tips for risk factor, ^∗^*P* < 0.05 in PRBD, daily LED, and the HAMD scores.

**Table 3:** Analysis of relative factors of RLS severity in PD patients.

Relevant factors	*r*_s_	*P* value
Sex	-0.054	0.807
Age at presentation	0.142	0.517
Age of PD onset	0.036	0.870
Duration of PD	0.438	0.036^∗^
Unilateral/bilateral involvement	0.199	0.056
UPDRS III	0.308	0.152
Hoehn and Yahr grade	0.376	0.077
PRBD	-0.199	0.362
MMSE score	-0.067	0.761
HAMD score	0.257	0.236
Daily LED	0.637	0.001^∗^

PD = Parkinson's disease; RLS = restless leg syndrome; UPDRS III = unified Parkinson's disease rating scale III; PRBD = probable rapid eye movement sleep behavior disorder; MMSE = minimum mental state examination; HAMD = Hamilton depression scale; daily LED = daily levodopa equivalent dose; *r*_*s*_ > 1 tips for positive correlation, ^∗^*P* < 0.05 between the severity of RLS in PD patients with duration of PD or daily LED.

## Data Availability

The data used to support the findings of this study are available from the corresponding author upon request.
